# The short-form revised Eysenck personality questionnaire: A Hindi edition (EPQRS-H)

**DOI:** 10.4103/0972-6748.57854

**Published:** 2009

**Authors:** Trayambak Tiwari, Anju L. Singh, Indramani L. Singh

**Affiliations:** Cognitive Science Laboratory, Department of Psychology, Banaras Hindu University, Varanasi - 221005, (U.P.), India

**Keywords:** EPQR - Short, Extraversion, Neuroticism, Psychoticism, Lie score

## Abstract

**Background:**

There is a growing consensus about the validity of human personality traits as important dispositions toward feelings and behaviors (Matthews, Deary, & Whiteman, 2003).

**Materials and Methods:**

Here we examine the reliability of the Hindi translation of the Eysenck Personality Questionnaire-Revised Short Form (EPQR-S; Eysenck, Eysenck, & Barrett, 1985), which consists of 48 items that assess neuroticism, extraversion, psychoticism, and lying. The questionnaire was first translated into Hindi and then back translated. Subsequently, it was administered to 202 students (78 men and 124 women) from Banaras Hindu University. The internal consistency of the scale was evaluated.

**Results:**

The findings provide satisfactory psychometric properties of the extraversion, neuroticism and lie scales. The psychoticism scale, however, was found to be less satisfactory.

**Conclusion:**

It can be proposed that due to satisfactory internal consistency scores, the EPQRS-H is a reliable scale for the measurement of various personality traits.

In its preliminary version, the Eysenck personality theory involved neuroticism-stability and extraversion-introversion dimensions; subsequently, the psychoticism dimension was added to the theory (Lewis *et al*., 2002). As the extraversion dimension represents sociality and impulsivity, individuals in this dimension were defined as enjoying social interactions, energetic, and preferring social situations to loneliness. It was proposed that the neuroticism dimension indicated emotional instability and reactiveness, and that individuals who score high on this dimension tend to be anxious, depressive, overly emotional, shy, and have low self-esteem. The psychoticism dimension highlights more bizarre personality characteristics, such as being distant, cold, insensitive, absurd, and unable to empathize with others (Eysenck& Eysenck, 1975).

Since the development of Eysenck personality theory, various measures were developed in order to assess the various personality traits. One of the consequences of this process has been a progressive increase in their length. The early Maudsley Medical Questionnaire (MMQ) contains 40 items (Eysenck, 1952), the Maudsley Personality Inventory (MPI) contains 48 items (Eysenck, 1959), the Eysenck Personality Inventory (EPI) contains 57 items (Eysenck& Eysenck, 1964a), the Eysenck Personality Questionnaire (EPQ) contains 90 items (Eysenck& Eysenck, 1975) and the Revised Eysenck Personality Questionnaire (EPQR) contains 100 items (Eysenck, Eysenck,& Barrett, 1985). This increase in length can be accounted for by the introduction of an additional dimension of personality within Eysenck’s scheme (Eysenck& Eysenck, 1976) and by the psychometric principle that greater length enhances reliability (Lord& Novick, 1968). Neuroticism and extraversion, especially, appear in most trait models of personality (Matthews *et al*., 2003). An important part of the validation of any trait-based model of personality and its associated measurement instrument is to investigate its applicability to other cultures. This tends to be done in two ways: emic and etic. Emic research typically uses the lexicon of the local culture to investigate the structure and content of the personality-related terms (Saucier& Goldberg, 2001). Etic research applies personality measures devised in one culture to new cultures and asks whether they show the same psychometric structure and reliability and validity (McCrae, 2001). A large amount of etic research has been completed on the Eysenck Personality Questionnaire. The research has been done mostly on the original 90-item EPQ. Generally, its psychometric structure has been well-reproduced in at least 34 countries (Barrett& Eysenck, 1984; Barrett, Petrides, Eysenck,& Eysenck, 1998).

Although all these questionnaires were reliable and valid there are, however, some practical disadvantages in using long tests. In particular, they caused certain clinical application problems due to their length. Therefore, the need for shorter personality scales resulted in shorter versions of the mentioned instruments. One of these shorter personality scales is the Eysenck Personality Questionnaire Revised - Short Form (EPQR-S; Eysenck *et al*., 1985). EPQR-S includes 48 items and 4 subscales: Extraversion (12 items), Neuroticism (12 items), Psychoticism (12 items), and Lie (12 items). The lie subscale is a control scale in which the whole scale is tested for social desirability bias. Eysenck *et al*. (1985) reported reliabilities for males and females respectively of 0.84 and 0.80 for neuroticism, 0.88 and 0.84 for extraversion, 0.62 and 0.61 for psychoticism, and 0.77 and 0.73 for the lie scale. The EPQR-S has now been used quite widely (Aleixo& Norris, 2000; Blagrove& Akehurst, 2001; Chan& Joseph, 2000; Chivers& Blagrove, 1999; Creed, Muller,& Machin, 2001; Francis, 1999; Francis& Wilcox, 1998; Glicksohn& Bozna, 2000; Glicksohn& Golan, 2001; Halamandaris& Power, 1999; Linton& Wiener, 2001; Martin& Kirkaldy, 1998; Robbins, Francis& Rutledge,1997).

In a cross-cultural study, Francis, Brown, and Philipchalk (1992) compared the psychometric properties of the EPQR-S in four English-speaking countries among a total of 685 undergraduate students, including 59 men and 153 women in England, 57 men and 92 women in Canada, 51 men and 81 women in the USA and 53 men and 139 women in Australia. According to this study the short form extraversion scale achieved alpha coefficients of 0.78, 0.83, 0.85 and 0.87 in the four samples. The short form neuroticism scale achieved alpha coefficients of 0.79, 0.80, 0.81 and 0.83 in the four samples. The lie scale performed less well than the extraversion and neuroticism scales, but proved to be adequate. The short form lie scale achieved alpha coefficients of 0.65, 0.66, 0.70 and 0.71. However, for the psychoticism scale, alpha coefficients were very low (0.33-0.52).

While the EPI, EPQ and EPQR were originally developed in England and then extended to other English-speaking areas, the cross-cultural extension of this field of personality research quickly led to the translation and testing of the instruments in non-English speaking environments (Barrett& Eysenck, 1984; Eysenck& Eysenck, 1983). For example, Francis and associates (Francis, Lewis,& Ziebertz, 2006) have developed the German edition of the EPQR-S. Similarly, Ivkovic *et al*. (2007) have developed and checked psychometric properties the Croatian edition of the EPQR-S.

Against this background, the aim of the present study was to examine the psychometric properties of the Hindi translation of the EPQR-S for Indian Hindi speaking college going adult population.

## MATERIALS AND METHODS

### Sample

Two hundred two (78 men and 124 women) Hindi speaking students studying in the Banaras Hindu University completed the Hindi translation of the EPQR-S. The age of the respondents ranged from 18 to 30 years with mean age of 22.27 years and SD of 2.37.

### Tool

#### Eysenck Personality Questionnaire Revised-Short Form (EPQR-S)

EPQR-Short (Eysenck, Eysenck& Barrett, 1985) is a self-reported questionnaire. It has 48 items, 12 for each of the traits of neuroticism, extraversion, and psychoticism, and 12 for the lie scale. Each question has a binary response, ‘yes’ or ‘no’. Each dichotomous item was scored 1 or 0, and each scale had a maximum possible score of 12 and minimum of zero.

#### Procedure

For the present study the questionnaire was translated into Hindi by a bilingual Indian national and then back-translated into English by a second bilingual Indian national in order to test for inaccuracies and ambiguities. Where there were inconsistencies in the retranslated English version, both translators were consulted as to the best possible solution. This content-based checking provided clear support for scoring the neuroticism, extraversion and lie scale items as suggested by Eysenck *et al*. (1985). After the content-based analysis the Hindi version of EPQR-S (here after referred as EPQRS-H) it was administered on all the participants (N=202) in order to examine its psychometric properties.

### Statistical analysis

The internal consistency of the four subscales of EPQRS-H was calculated using Chronbach’s alpha method (Cronbach, 1951).

## RESULTS

Table 1 presents the Corrected Item-Total Correlations for each of the four subscales of the EPQRS-H (i.e., extraversion, neuroticism, psychoticism and the lie scale). The reliability of the extraversion, neuroticism, psychoticism and lie score subscales were found to be 0.766; 0.772; 0.238; 0.624, respectively. The results indicated that none of the items were psychometrically poor. The corrected item-total correlation ranged from 0.201 to 0.538 for extraversion, from 0.196 to 0.556 for neuroticism, from 0.109 to 0.449 for lie scale and from —0.020 to 0.284 for psychoticism subscale of EPQRS-H. Moreover, none of the ‘alpha-if item deleted’ values exceeded the overall alpha except for two items of psychoticism subscale and one item of lie score subscale which were when deleted exceeded the overall alpha level of that subscale. When those items were thoroughly checked by the experts they suggests that the items are congruent with remaining items of that factor therefore these items should not be dropped from the scale. The psychometric analyses further shows that the neuroticism, extraversion and lie subscales perform well in this sample, but not the psychoticism subscale.

**Table 1:** Results of item analysis for the four subscales of the EPQRS-H

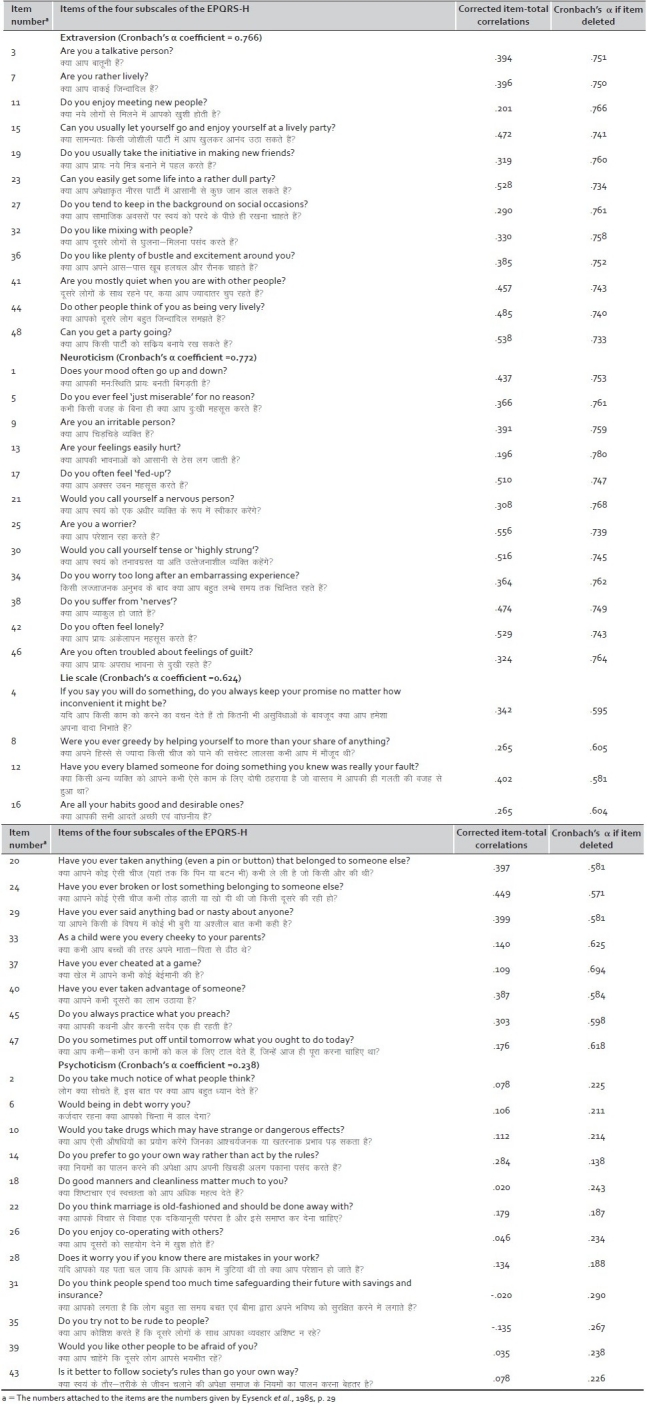

## DISCUSSION

The present study was aimed to evaluate the internal consistency of the Hindi translation of the EPQR-S. Both the extraversion and the neuroticism subscales of the Hindi translation of the EPQR-S achieved satisfactory alpha coefficients well in excess of 0.7, the level recommended by Kline (1993). The lie scale with an alpha coefficient of 0.624 is also reached very close to Kline’s criterion of 0.7. The psychoticism scale, however, performed poorly with an alpha coefficient of only 0.238.

When evaluated in general, it can be proposed that due to satisfactory internal consistency scores, the EPQRS-H is a reliable scale for the measurement of various personality traits. With regards to low internal consistency coefficients for the psychoticism subscale, various studies conducted in other countries also found the similar results (Francis *et al*., 1992, 2006; Ivkovic *et al*., 2007; Katz and Francis, 2000; Lewis *et al*., 2002). It was therefore concluded that the low alpha score of psychoticism scale was not related to the Hindi translation (that this subscale can be problematic). At the same time these data emphasize the need for further research and development to produce a more reliable short index of psychoticism.

The fact that the sample of the study included college participants from an Indian university supports the psychometric properties (i.e., reliability) of EPQRS-H. It would be beneficial to repeat the study with heterogeneous sample and to examine its discriminative value with clinical population. An attempt should also be made in future to investigate the temporal consistency and factorial validity of the EPQRS-H with larger sample.
